# Treatments of traumatic neuropathic pain: a systematic review

**DOI:** 10.18632/oncotarget.16917

**Published:** 2017-04-07

**Authors:** Chenglun Yao, Xijie Zhou, Bin Zhao, Chao Sun, Keshav Poonit, Hede Yan

**Affiliations:** ^1^ Department of Orthopedics (Division of Plastic and Hand Surgery), The Second Affiliated Hospital and Yuying Children’s Hospital of Wenzhou Medical University, Wenzhou, China

**Keywords:** traumatic neuropathic pain, neuroma, treatment modality, pain management

## Abstract

Traumatic neuropathic pain caused by traumatic neuroma has long been bothering both doctors and patients, the mechanisms of traumatic neuropathic pain are widely discussed by researchers and the treatment is challenging. Clinical treatment of painful neuroma is unclear. Numerous treatment modalities have been introduced by experts in this field. However, there is still no single standard recognized treatment.

Different forms of treatments have been tested in animals and humans, but pharmacotherapies (antidepressants, antiepileptics) remain the basis of traumatic neuropathic pain management. For intractable cases, nerve stump transpositions into a muscle, vein or bone are seen as traditional surgical procedures which provide a certain degree of efficacy. Novel surgical techniques have emerged in recent years, such as tube guided nerve capping, electrical stimulation and adipose autograft have substantially enriched the abundance of the treatment for traumatic neuropathic pain.

Several treatments show advantages over the others in terms of pain relief and prevention of neuroma formation, making it difficult to pick out a single modality as the reference. An effective and standardized treatment for traumatic neuropathic pain would provide better choice for researchers and clinical workers.

In this review, we summarized current knowledge on the treatment of traumatic neuropathic pain, and found a therapeutic strategy for this intractable pain. We tried to provide a useful guideline for choosing the right modality in management of traumatic neuropathic pain.

## INTRODUCTION

Peripheral neuroma caused by injuries and surgical procedures can result in traumatic neuropathic pain, functional impairment and psychological distress, severely decreasing the quality of life [1]. A traumatic neuroma is a tangle of neural fibers and connective tissue that develops following nerve injury. It usually presents as a firm, oval, whitish, slowly growing, palpable and painful nodule, not larger than 2 cm. It may be associated with paresthesia over the injured area [2]. Painful hypersensitivity to normal light tactile stimuli (dysesthesia) or a neuralgic pain with the presence of a typical trigger point in the area of a neuroma may be a prominent feature. Traumatic neuropathic pain can cause the patient to feel burning, stabbing, raw, gnawing or sickening sensations. [2].

Numerous techniques for neuroma prevention and treatment have been proposed, including therapeutic massage [3], electrical stimulation [4], lipofilling [5], transposition of the proximal stump into the muscle, bone or vein [6–11], and neural capping with synthetic or biological materials [12–16]. The diversity of treatment modalities in use reflects the currently frustrating clinical prognosis and high failure rates. Currently, the treatment of traumatic neuroma is challenging with varied symptoms among patients, some of which are usually resistant to common analgesics, and a standardized management is highly demanded in clinical practice.

In this review, we aimed to summarize current knowledge on the treatment of traumatic pain and generate a therapeutic strategy for this intractable clinical challenge, hoping to provide a useful guideline for choosing the appropriate modality in management of different kinds of traumatic neuropathic pain.

## RESULTS

An initial search returned a total of 123 papers, published between 1994 and 2016. After abstract and reference review, additional useful references added a total of 36 articles that were selected. In the literature, many treatment modalities of post-traumatic neuropathic pain have been described, all of which were divided into four categories: classical surgical procedures, emerging surgical procedures, medication and other treatments for traumatic neuroma. An overview on the main treatment modalities for painful neuroma is provided. Each typical treatment is discussed focusing on their advantages and disadvantages. Data on different treatment modalities are summarized in Tables 1 to 4, respectively. And a treatment strategy is generated based on our findings in this review (Figure 2).

**Table 1:** Traditional surgical procedure for traumatic neuroma

First Author	Year	Patient mean Age(year)	Nerve	Type of Injury	Treatment	Follow up (month)	Evaluation Methods	Treatment Outcome
Evans et al [6]	1994	43	Median nerve	Carpal tunnel release surgery	Implantation into the pronator quadratus	19	-Questionnaire-PE	92.3% (12 of the 13) patients had subjective improvement in pain.
Yuksel et al [36]	1997	33	Digital nerve	Amputation	Epineural ligatures, flaps and grafts	6	VAS	Pain disappeared 2 months following surgery.
Sood et al [8]	1998	39.2	Nerves of the palm and the dorsum of the hand	Surgery	Resection and relocation into the pronator quadratus	4 and 24	-Questionnaire-PE	No patient had spontaneous pain.
Stahl et al [9]	2002	52	Medial antebrachial cutaneous nerve	Direct elbow trauma	Resection and implantation into the triceps muscle.	6	-VAS-Dynamometer	9 patients improved to excellent and good pain scores.
Koch et al [10]	2004	44.5	-Saphenous nerve-Sural nerve-Femoral nerve-Medial nerve	-Surgery	Transplantation into a vein	17	-Questionnaire-PE	Immediate relief of pain
Balcin et al [11]	2009	46	-Sural nerve-Foot dorsal cutaneous nerve-Peroneal nerve-Saphenous nerve	Trauma	Translocation into muscle and vein	3 and 12	-VAS-Questionnaire	Significant improvement in pain in the vein group
Pet et al [7]	2014	N/A	-Median nerve-Radial nerve-Ulnar nerve-Musculocutaneous nerve-Thoracodorsal nerve-Tibial nerve-Peroneal nerve	Amputation caused by trauma and surgery	Implantation into muscle	8 to 60	VAS	11 of 12 patients (92%) were free of pain
Hanna et al [1]	2016	12.2	-Digit nerve-Posterior interosseous, intercostal nerve-Ulnar nerve-Superficialperoneal nerve	-Trauma-Surgery-Fracture	-Coaptation-Capping-Burial into muscle-Allograft-Ligation-Division	12	-Questionnaire-PE	Complete resolution of symptoms.

PE=physical examination, VAS=visual analog scale.

**Table 2:** Novel surgical procedures for traumatic neuroma

First Author	Year	Patient mean Age(year)	Nerve	Type of Injury	Treatment	Follow up(month)	Evaluation Methods	Treatment Outcome
Krishnan et al [18]	2005	45.1	Digital nerve	-Trauma-Sugery	-Resection-Neurolysis-Vascularized fascial-Fasciocutaneous-Flap	16.6	QVAS	Mean QVAS values (pain now/typically/at its best/at its worst) changes from 6.5/6.5/4.7/7.9 to 0.3/0.4/0/0.9.
Peterson et al [20]	2006	39.2	Radial sensory nerve	-Trauma-Sugery	-Neuroma excision-Neurolysis-Interposition of acellular dermal matrix allograft	12 to 25	VAS	All patients reported a subjective decrease in pain level.
Kakinoki et al [19]	2008	46	Palmar digital nerve	Trauma	Cover the tips of digits using skin islands along with subcutaneous nerves.	17	-Grading system-SWMFT	The neuroma-related symptoms disappeared completely in 6 patients.
Thomsen et al [12]	2010	30	-Digital nerve-Common digital nerve	Trauma	Coverage using 20 mm or 30 mm collagen tubes.	11.8	-s2PD-SWMFT-CISS-The Quick-Dash	All the patients were satisfied.
Gennady Gekht et al [23]	2010	17	Medial nerve	Trauma	Minimally invasive neurectomy	3 and 7	-VAS-ODI	Complete resolution of pain.
Ulrich et al [5]	2011	32.5	Vulvar nerve	Episiotomy	-Liposuction-Lipofilling	7 and 9	VAS	The episiotomy scar was eutrophic and the Tinel sign was negative.
Martins et al [17]	2015	30.3	Digital nerve	Finger amputationSurgery	Interdigital direct neurorrhaphy	28.3	-VAS-DASH	Improvement in upper limb functionality and pain. VAS and DASH scores improved by 29.8% and 55.5 %.
Economides et al [16]	2016	N/A	-Tibial nerve-Sciatic nerve	AmputationSurgery	Transfemoral amputation and nerve management	2 and 6	VAS	Mean VAS scores as well as rates of neuroma and PLP/PS were significantly lower.

QVAS=quadruple visual analog scale, VAS=visual analog scale, SWMFT=Semmes-Weinstein monofilament test, s2PD=static two point discrimination, CISS=cold Intolerance symptom severity, ODI=Oswestry disability index, DASH=the disabilities of the arm, shoulder and hand score.

**Table 3:** Medication for traumatic neuroma

First Author	Year	Patient mean Age(year)	Nerve	Type of Injury	Treatment	Follow up(month)	Evaluation Methods	Treatment Outcome
Rizzo et al [25]	1997	63	Ilioinguinal nerve	Herniorrhaphy	4 to 5 mg/ml of carbamazepine (Systemic)	Continuous	-Questionnaire-PE	Significant relief from symptoms.
Dahl et al [31]	2008	25.8	-Fibular nerve-Tibial nerve	Amputation due to warcraft and a motor vehicle accident	Injections of etanercept (Local)	3	VAS	83.3% (5 of the 6) patients had significant improvements in pain.
Correa et al [28]	2010	41.4	-Upper extremity-Lower extremity-Trunk	-Burns-Skin degloving-Orthopedic surgery	5% lidocaine, Co-analgesics, and concomitant drugs (Local)	1 month to 2.8 years	-NRS-DN4 questionnaire- Pain area measurement	Nineteen patients (69%) showed functional improvement.
Touchette et al [29]	2011	67	Never of tongue	Surgery	Injections of alcohol with 2% lidocaine (Local)	4	Questionnaire	16% alcohol brought long-lasting relief of pain.
Singh et al [26]	2012	37	-Inferior alveolar nerve-Infra orbital nerve	-Trauma-Surgery	Tablet pregabalin(Systemic)	3	VAS	Complete relieve in pain.
Climent et al [33]	2013	58.19	-Second intermetatarsal space-Thirdintermetatarsal space	-Trauma-Surgery	Onabotulinumtoxin A(Local)	1 and 3	-VAS-FHS-Questionnaire	12 patients (70.6 %) reported an improvement in their pain.
Thomson et al [27]	2013	53	The second or third inter-metatarsal spaces or toes	-Trauma-Surgery	-Methylprednisolone-lignocaine (Local)	3	-VAS-Questionnaire	Significantly better.
Backryd et al [30]	2015	56	Inferior alveolar nerve	-Trauma-Surgery	Ziconotide(Local)	Hourly	VASPI	Pain intensity changed significantly over time (0–6 h).

PE=physical examination, VAS=visual analog scale, NRS=numeric rating scale, ND4=douleur neuropathique 4 questions, FHS=foot health status, VASPI=visual analogue scale pain intensity.

**Table 4:** Other treatment strategies for traumatic neuroma

First Author	Year	Patient mean age(year)	Nerve	Type of Injury	Treatment	Follow up(month)	Evaluation Methods	Treatment Outcome
Faith et al [3]	2012	25	Foot dorsal cutaneous nerve	-Trauma	Six session of massage therapy	Continuous	-VAS-Questionnaire	VAS score changed from 5 to 0.
Stevanato et al [4]	2014	46	-Median nerve-Radial nerve	-Trauma-Surgery	A quadripolar electrode lead was placed into the axillary cavity.	6 and 12	-QST-NRS-Questionnaire	All patients experienced pain relief within a few minutes

VAS=visual analog scale, QST=quantitative sensory testing, NRS=numeric rating scale.

**Figure 1:**
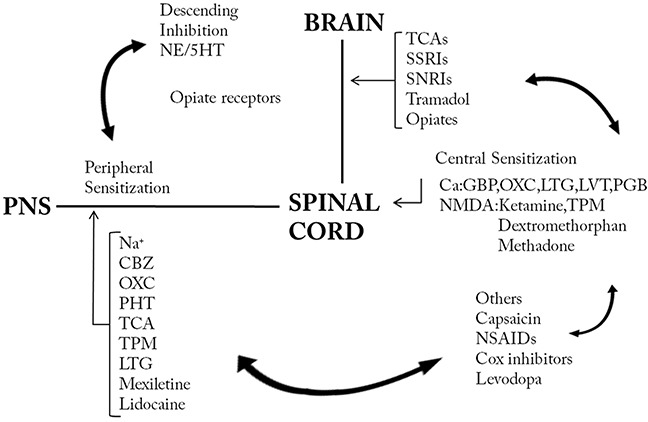
Mechanistic approaches of treatment in neuropathic pain CBZ=carbamazepine, Cox=cyclooxygenase, 5HT=5-hydroxytryptamine, GBP=gabapentin, LTG=lamotrigine, LVT=levitiracetam, NE=norepinephrine, NMDA=N-methyl-D-aspartate, NSAID=non-steroid anti-inflammatory drug, OXC=oxcabazepine, PHT=phenytoin, PNS=peripheral nervous system, SNRI=selective serotonin norepinephrin reuptake inhibitor, SSRI=selective serotonin reuptake inhibitor, TCA=tricyclic antidepressant, TPM=topiramate.

**Figure 2:**
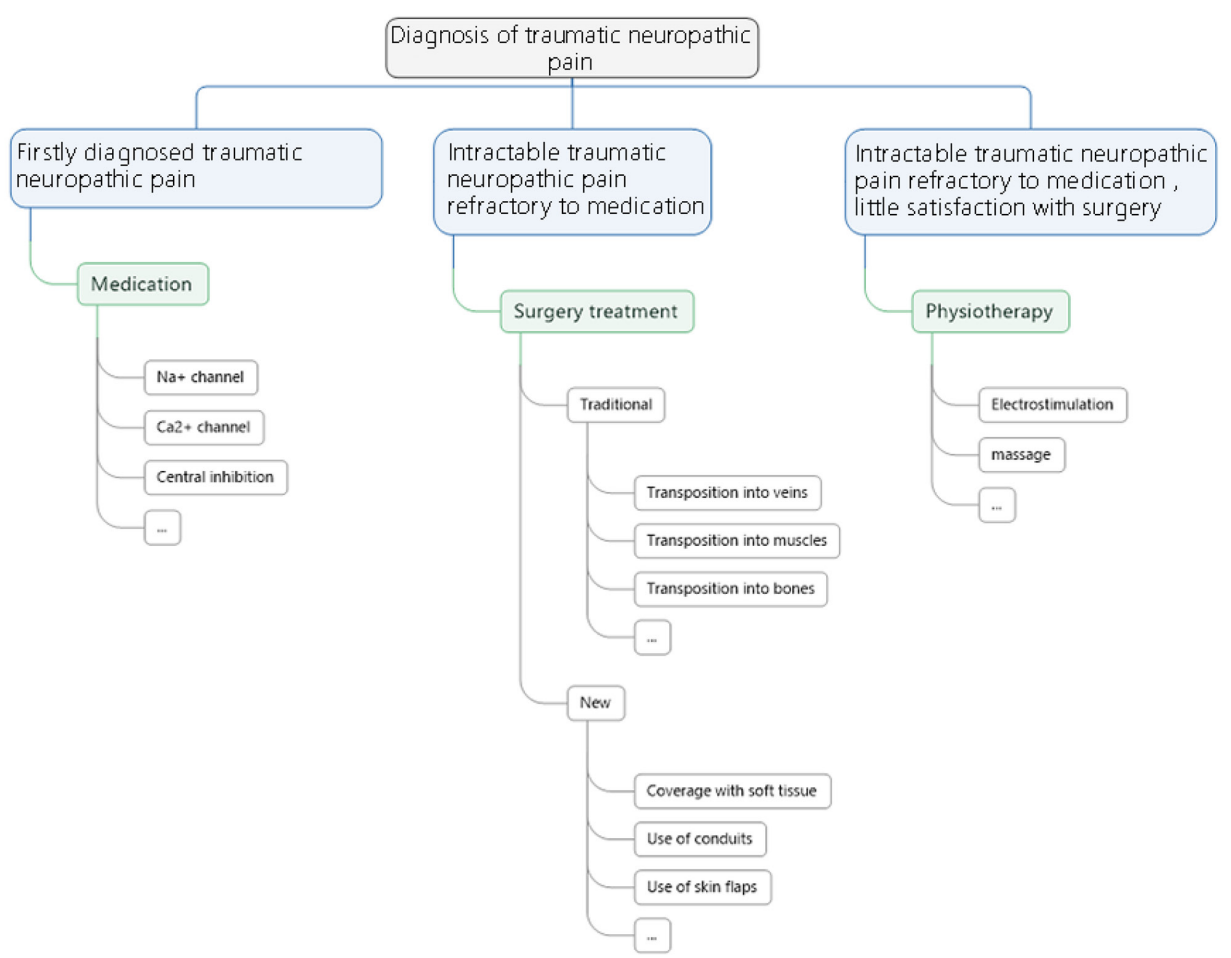
A flowchart of treatment principles for neuropathic pain

### Treatment strategies

#### Classical surgical procedures

Classical surgical treatments for traumatic neuropathic pain include resection of the neuroma, neurorrhaphy and implantation of the nerve stump into muscles, bones and veins. The key of this treatment modality is to remove external pressure thus preventing simultaneous proliferation of nerve and fibrous connective tissue and to maintain the dynamic balance of the microenvironment surrounding the injured nerve. Reinnervation of the distal target or translocation from the distal end organ to a site with minimal potential for further stimulation is the usual treatment [11]. Evans [6] et al. combined resection of the neuroma with implantation of the palmar cutaneous branch of the median nerve (PCB) into the pronator quadratus muscle, which allowed the nerve to be isolated from wrist motion and denervated skin. The efficacy of this treatment modality was examined, follow-up data were obtained on all patients at a mean of 19 months after surgery, and subjective improvement in pain was reported in 12 of the 13 patients [6]. Koch and his team suggested the possibility of inhibiting the formation of painful neuromas by nerve transposition into a vein [10]. Balcin compared the two methods of surgical treatment for painful neuroma by transposition of the nerve stump into an adjacent vein or muscle. Compared to their pre-operative levels in the muscle group three and 12 months after surgery, translocation into a vein led to reduced intensity and evaluative levels of pain, as well as improved sensory as assessed by visual analogue scale and McGill pain score. This was associated with an increased level of activity and improved function. Transposition of the nerve stump into an adjacent vein is preferred to relocation into a muscle [11]. Recently, Martins analyzed data from seven patients using interdigital neurorrhaphy for treatment of digital neuromas. All patients showed some temporary pain relief after nerve block, and therapeutic results were observed during later follow-up. Their findings indicated that interdigital neurorrhaphy achieved satisfactory improvement of symptoms and hand dysfunction [17]. Classical surgeries are mostly used in clinical practice and they can indeed provide a certain treatment efficacy. However, all these treatment modalities do have certain respective limitations in practice, for instance, the method of nerve transposition into a vein requires the existence of a suitable vein for the nerve stump to relocate in. The technique like interdigital neurorrhaphy is technically demanding and sometimes no corresponding nerve to be sutured with.

#### Novel surgical procedures

In recent years, several new surgical strategies for traumatic neuropathic pain have attracted interest. The use of soft tissue [5, 18–21] and the use of conduits [12, 15, 16, 21] have showed great potential in the treatment of traumatic neuropathic pain. Research has shown that myofibroblasts are highly expressed in the neuroma, it is speculated that myofibroblasts may contribute to pain by contracting the collagen matrix around the sensitive non-myelinated fibers that grow astray to form a neuromatous bulging [18]. Krishnan [18] concludes that vascularized soft tissue coverage of painful peripheral nerve neuromas can be an effective, but also a complex method of treatment. All kinds of nerve conduits have been popularized in the treatment of nerve defects for years [22], and they have also been introduced in the management of painful neuromas [12]. A research conducted by Marcol [21] et al. found microcrystallic chitosan conduit could efficiently prevent the neuroma formation and traumatic neuropathic pain in a rat sciatic nerve model. Clinically, Peterson [20] et al. also achieved satisfactory outcomes using an acellular dermal matrix conduit in treatment of traumatic neuropathic pain at the wrist. The key of these treatment modalities is to cover or protect the injured nerve stump from physical and chemical stimulation, thus eliminating the external stimuli and keeping the injured nerve stump under a relatively stable environment. Similarly, Gennady [23] demonstrates a minimally invasive neurectomy to treat painful medial branch neuroma, which poses little damage to surrounding tissue and indirectly provides good prognosis. In our previous study, we used aligned nanofiber conduits in the management of painful neuromas in rat sciatic nerves. We found that aligned nanofiber conduits can significantly facilitate linear nerve regeneration, inhibit neuroma growth, and reduce traumatic neuropathic pain after neurectomy [24]. Synthetic conduits can be a promising field in the treatment of traumatic neuropathic pain, as the material science evolves.

### Medication

Known as a conservative treatment modality, medication is usually the initial choice for patients with traumatic neuropathic pain. The mechanisms of different medications were summarized in Figure 1. Rizzo [25] found carbamazepine, a representative of sodium channel blocker, was effective in the treatment of painful paresthesias in the territory of the ilioinguinal nerve. In addition, pregabalin, a voltage dependent calcium channel blocker, was also introduced clinically in the treatment of painful neuromas with good results [26]. In terms of local medication, glucocorticoids have been utilized for years. Thomson [27] uses methylprednisolone injections for the treatment of Morton neuroma, which was considered as a kind of painful traumatic neuroma between adjacent metatarsal heads. The corticosteroid injection group achieved significantly better Foot Health Thermometer scores compared with the control group at both one and three months after injection [27]. Nevertheless, local injections of steroids may cause tissue atrophy and long term results of this treatment modality needs to be further examined. Meanwhile, local anesthetics are also a common choice for traumatic neuropathic pain; Correa-Illanes [28] found that 5% lidocaine medicated plaster has a profound efficacy in traumatic neuropathic pain in scars. After treatment, the numerical rating scale (NRS) was reduced by 58.2% ± 27.8%. The average painful area was reduced by 72.4% ± 24.7%. Nineteen patients (69%) showed functional improvement following treatment. A case reported by Touchette [29] used serial alcohol injections for the treatment of painful traumatic neuroma of the tongue; the patient obtained satisfying outcomes when the alcohol concentration was increased to 16%. However, local alcohol injections may cause damage or death of nearby nerves and tissue; in addition, it may also cause infection, pain, weakness, dysarthria, and poor symptom resolution. Additionally, invasive method of analgesics, intrathecal infusion for example, is also reported. Backryd [30] applied ziconotide, which is an atypical analgesic agent for the amelioration of severe and chronic pain, for intrathecal bolus injections in the treatment of postoperative/posttraumatic neuropathic pain in patients refractory to conventional treatment. Precautions should be taken because ziconotide has several neurological adverse events, for instance, dizziness, ataxia, abnormal gait, nystagmus, or nausea; it also has a narrow therapeutic window which cannot be ignored. Cytological level medication therapy including tumor necrosis factor (TNF) inhibitor [31], nerve growth factor (NGF) inhibitor [32], cytotoxin [33] and the potential function of alpha smooth muscle actin [34] has also been reported in literature.

### Other treatments

Other treatments for traumatic neuropathic pain are mainly physiotherapy. Therapeutic massage is advised by Faith [3], and which is preferred for the treatment of Morton’s neuroma. Electrical stimulation is another common conservative treatment choice. In Stevanato’s [4] et al. study, they use an implanted peripheral nerve stimulator applied directly to the nerve branch involved into the axillary cavity. A quadripolar electrode lead was placed directly on the sensory peripheral branch of the main nerve involved, proximally to the site of lesion, into the axillary cavity. According to their “gate-control” theory, the peripheral nerve stimulation produces direct electrical effects by recruitment of primary afferent A-beta and A-delta fibres that project at the spinothalamic tract and dorsal columns and A-alpha fibres that cause segmental inhibition through presynaptic inhibitory interneurons. It can be considered as a reasonable treatment of patients suffering from otherwise intractable painful neuropathies of the upper arm.

### Evaluation methods

Although there are several different evaluation methods referred in the papers, the most commonly used measurement tool is visual analog scale (VAS). It is the historical gold-standard evaluation method, but it needs to be correctly tested. Questionnaire is the second commonly used tool in literature. VAS provides a direct quantitative measure of functional recovery, while questionnaire can indicate the patient’s subjective sense over the related region. Histology and electrophysiological evaluation are difficult to operate on human, but common in animal experiments. These tools can directly reflect patients’ pain status and allow a more accurate evaluation of pain. As for experimental researches, behavioral study is the indirect way to assess the pain status of an animal, histology and other immunohistochemistry analysis can be useful tools to evaluate some certain proteins associated to pain status.

## DISCUSSION

A practical and standardized treatment modality is desirable, as it would allow a better comparison of results from different studies. However, this review points out great differences in published treatments, especially between choices of surgical procedures and medicines. Several of these treatments are better regarding to a specific aspect of prevention and efficacy. However, considering the different injured nerves and different kind of symptoms, it is unlikely that a single treatment modality could be of reference for all. Additionally, in our previous study, we found that the expression of alpha smooth muscle actin (α-SMA) may contribute to neuroma-associated pain either as a direct cause of pain or an indirect marker of existence of local mechanical stimuli [35]. The potential role of α-SMA in the pathobiology of traumatic neuropathic pain might be considered as a treatment option in clinical practice.

Furthermore, due to the great differences in all aspects of the different treatment modalities (nerves, types of injury and evaluation methods), it is not possible to statistically compare the reviewed studies and their results; thus, evidence-based indications for the standardization of neuroma treatment modalities cannot be extrapolated from this review. Nevertheless, based on the advantages and popularity of available treatments, we propose our suggestions for standardization and initially generate a pilot treatment strategy for the traumatic neuropathic pain.

Treatment modality: Medication should be doctors’ first choice for patients who develop traumatic neuropathic pain for the first time, it also should be advised for prevention postoperatively. For patients who undergo intractable traumatic neuropathic pain, which is refractory to medication, surgical procedure is advised. As for which kind of surgery should be performed, it depends on which particular nerve is injured. For example, neuroma of palmar cutaneous branch (PCB) of the median nerve can be transpositioned into the pronator quadratus, while the traumatic neuropathic pain in an amputated finger is suggested to be treated with interdigital neurorrhaphy. In general, resection of the existing neuroma is strongly advised for all the patients with traumatic neuropathic pain. Transposition and relocation of the nerve stump into a biological tunnel away from partial compression, for instance, muscles, bones and veins are necessary to assure long term efficacy. Artificial conduits can be used under the circumstances that no suitable biological tunnel can be found. Soft tissues treatment, for instance, flaps and lippofilling can provide better prognosis for those patients with large scale of tissue defect and deep soft tissue injury. Lastly, for patients with intractable traumatic neuropathic pain refractory to medication and refuse any surgical treatment or have little satisfaction with surgery, physiotherapy as electrostimulation and massage may also be good choices. The treatment modality is summarized in a flowchart as shown in Figure 2.

## MATERIALS AND METHODS

A systematic electronic search was performed in the PubMed, ISI web of science, EMBASE, Science Direct database combining ‘treatment’, ‘therapy’, ‘management’, ‘surgery’ and ‘neuroma’, ‘traumatic’, ‘pain’ as search terms (all fields). Studies in languages other than English and those not associated with treatments of traumatic neuropathic pain in clinical practice were excluded based on abstract review. Studies proposing a treatment modality or a modification of an existing treatment modality for neuromas were then selected based on a full-text article review, whereas those using animals for experimental researches were excluded. References of selected articles were evaluated to identify further relevant articles. Selected articles were reviewed, analyzing the treatment modalities, the evaluation methods and the treatment outcomes of the neuromas.

This study was funded by the National Natural Science Foundation of China (81571185).
